# Recommendations for use of topical inhalant budesonide in COVID-19: A Position Paper of the German Society for Applied Allergology (AeDA) and the German Society of Oto-Rhino-Laryngology, Head and Neck Surgery (DGHNO)

**DOI:** 10.1007/s00106-021-01070-9

**Published:** 2021-07-16

**Authors:** Ludger Klimek, Roland Buhl, Thomas Deitmer, Stefan Plontke, Wolfgang Wehrmann, Hans Merk, Johannes Ring, Sven Becker, Ärzteverband Deutscher Allergologen (AeDA), Sven Becker, Ludger Klimek, Hans Merk, Johannes Ring, Wolfgang Wehrmann, Deutsche Gesellschaft für Hals-Nasen-Ohren-Heilkunde, Kopf- und Hals-Chirurgie (DGHNO-KHC), Thomas Deitmer, Stefan Plontke

**Affiliations:** 1 Zentrum für Rhinologie und Allergologie Wiesbaden, Wiesbaden, Germany; 2 Schwerpunkt Pneumologie, III. Medizinische Klinik und Poliklinik, Universitätsmedizin Mainz, Mainz, Germany; 3 Deutsche Gesellschaft für Hals-Nasen-Ohren-Heilkunde, Kopf- und Hals-Chirurgie, Bonn, Germany; 4 Klinik und Poliklinik für Hals-Nasen-Ohren-Heilkunde, Kopf- und Hals-Chirurgie, Martin-Luther-Universität Halle-Wittenberg, Halle, Germany; 5 Dermatologische Gemeinschaftspraxis Wehrmann, Münster, Germany; 6 Abteilung Dermatologie & Allergologie, RWTH Aachen, Aachen, Germany; 7 Haut- und Laserzentrum an der Oper, Munich, Germany; 8 Klinik für Hals-, Nasen- und Ohrenheilkunde, Kopf- und Halschirurgie, Eberhard Karls Universität Tübingen, Elfriede-Aulhorn-Str. 5, 72076 Tübingen, Germany

Inhalational glucocorticosteroids (ICS) are considered standard therapy for inflammatory diseases of the airway mucosa, such as allergic rhinitis (AR), chronic rhinosinusitis (CRS), or bronchial asthma (A; [1, 2]). Chronic rhinosinusitis, AR, and A are among the most common inflammatory diseases, and chronicity is often associated with epithelial damage and tissue destruction that can promote viral infections [1, 2].

Bronchial asthma is an important comorbidity of AR and CRS. Deterioration in the control of AR and CRS may promote asthma exacerbations [3–7].

After indications were initially published in the current SARS-CoV‑2 pandemic that “cortisone preparations” could increase the risk of contracting COVID-19 or cause a more severe course of the disease and thereby massively unsettled numerous patients with AR, CRS, and asthma. Position papers of the German and European societies pointed out the necessity of continuing therapy with topical GCS very early in the pandemic [1, 2]. Accordingly, ICS and nasal GCS (nGCS) are effective in the treatment of mucosal inflammation of the upper and lower respiratory tract and represent the standard therapy for these conditions [8–11].

No evidence exists to suggest that use of nGCS and ICS triggers an increased risk of SARS-CoV‑2 infection or a more severe course of COVID-19-disease. Therefore, adults and children with AR, CRS, and A should consistently and regularly take their prescribed nGCS and ICS at the individually prescribed dose and not change or even discontinue them without consulting the treating physician [1, 2]. Here, we also point out that effective anti-inflammatory control of the upper and lower airways by topical GCS is a good protection against viral-triggered exacerbations for these patients according to current medical knowledge [1, 2]. From today’s perspective, there are sufficient data that patients with chronic inflammatory airway disease should receive guideline-based pharmacologic treatment in the context of the COVID-19 pandemic, including nGCS, ICS, and biologic therapies if needed [12, 13]. The aforementioned topical GCS include budesonide.

## Study on the use of budesonide in COVID-19

In the latest issue of *The Lancet Respiratory Medicine*, Ramakrishnan et al. published a hypothesis-generating study on the use of inhaled budesonide versus “standard therapy” in patients with early COVID-19 [14]. The authors conducted a phase 2, open-label, randomized controlled, parallel-group study to compare the use of inhaled budesonide (1600 µg/day) with standard therapy in patients with symptomatic COVID-19 established in the past 7 days [14]. The authors concluded that this treatment regimen may be the first cost-effective and readily available therapeutic intervention for early-stage COVID-19. They mention that their data may also represent a potentially effective treatment for preventing long-term and persistent COVID-19 symptoms.

## Evaluation of the study data

In our opinion, the above statements [14] are not supported by the data presented. This was an open-label study in which patients and physicians were informed about the type of therapy. Placebo effects of ICS in bronchial asthma have been described at a rate of 21–46%, especially when subjective outcome parameters are used as evaluation criteria (outcome parameters; [15]).

The effects observed in this study, including the primary endpoint (COVID-19-related outpatient presentation or hospitalization) and secondary endpoints (such as time to clinical improvement subjectively perceived by patients) may have been influenced by the subjective perceptions of affected patients and their treating physicians [16].

Objective measures such as blood oxygen saturation, body temperature, spirometry findings, and quantitative viral load with SARS-CoV‑2 were used as additional secondary endpoints in the study, but the results did not differ between groups. The study was small and included only 146 participants—73 were randomized to the “standard therapy” and 73 to the budesonide group.

Ramakrishnan et al. hypothesized that early administration of inhaled budesonide would reduce the likelihood of needing urgent medical care and shorten the time to recovery for patients with COVID-19 treated early [14]. Given the evidence from this and other studies, this interpretation of the data remains speculative.

The primary endpoint was met in 11 (15%) control patients and two (3%) budesonide patients (*p* = 0.009). Time to clinical improvement was 1 day shorter in the budesonide-treated patients (7 vs. 8 days, *p* = 0.007). Fever occurred in 2% of the budesonide patients in the 14 days after study entry compared with 8% of the control patients (*p* = 0.051). Adverse effects occurred in five patients (7%) of the budesonide group.

This fits with the interim results of a still-ongoing larger phase III trial of 2617 patients, also testing inhaled budesonide therapy for acute SARS-CoV‑2 infection [17]. This showed a 3-day shorter time to self-reported clinical improvement of symptoms, but the small effect on COVID-19-related hospitalizations or deaths (budesonide: 59/692 [8.5%] vs. 100/968 [10.3%] patients) was not statistically significant.

## Discussion

The German societies AeDA (German Society for Applied Allergology), DGP (German Respiratory Society), DGAKI (German Society for Allergology and Clinical Immunology), and DGHNO (German Society of Oto-Rhino-Laryngology, Head and Neck Surgery) together with international organizations such as ARIA (Allergic Rhinitis and Its Impact on Asthma Initiative), EAACI (European Academy of Allergy and Clinical Immunology), and GAL^2^EN (Global Allergy and Asthma European Network) emphasized the need for continuation and consistent use of ICS in patients with inflammatory airway diseases early in the pandemic [12, 13]. Similar recommendations were also made for other allergic diseases during the pandemic [18–20].

The available study results on inhaled budesonide therapy suggest that a somewhat shorter symptom duration can be achieved when high-dose inhaled budesonide therapy is started within a few days after the onset of COVID-19 disease. A higher-grade clinical effect cannot be demonstrated on the basis of current study data.

A cautious interpretation of these data is all the more warranted given that an updated interim analysis of data from a larger phase III trial that included 2617 participants with risk factors for unfavorable outcomes with COVID-19 did not show such favorable results [17]—inhaled budesonide shortened time to self-reported recovery by a median of 3 days, but did not meet the primary outcome parameter (predefined superiority threshold for the probability that COVID-19 hospitalizations/deaths were lower in the budesonide group compared with standard therapy): budesonide: 59/692 (8.5%) vs. standard therapy: 100/968 (10.3%) [17].

Recent data provide further evidence that patients with different asthma endotypes (type 2 asthma vs. non-type 2 asthma) have different risk profiles with respect to SARS-CoV‑2 infection, development of COVID-19, and progression to severe COVID-19 disease [21].

Effective measures to contain the pandemic, on the other hand, are restrictions on social life, especially to protect vulnerable patient groups and to maintain a functioning healthcare system. In agreement with the German Robert Koch Institute (RKI) and the World Health Organization (WHO), we recommend preventive measures in the current pandemic situation, such as [22–25]:

- Keeping a distance of at least 1.5–2 m from other people
- Adherence to general hygiene measures, such as regular hand disinfection/regular hand washing for at least 30 s, avoid touching mucous membranes with hands
- Minimizing social contact
- Limiting personal patient contacts to what is absolutely necessary
- Wearing personal protective clothing
- Regular surface disinfection, especially door handles, etc.
- Vaccination campaigns that are as rapid and comprehensive as possible

## Declarations

For this article no studies with human participants or animals were performed by any of the authors. All studies performed were in accordance with the ethical standards indicated in each case.

The supplement containing this article is not sponsored by industry.
